# *Prunus mume* and *Lithospermum erythrorhizon* Extracts Synergistically Prevent Visceral Adiposity by Improving Energy Metabolism through Potentiating Hypothalamic Leptin and Insulin Signalling in Ovariectomized Rats

**DOI:** 10.1155/2013/750986

**Published:** 2013-11-10

**Authors:** Byoung-Seob Ko, Da Sol Kim, Suna Kang, Jin Ah Ryuk, Sunmin Park

**Affiliations:** ^1^Korea Institute of Oriental Medicine, 1672 Yoosungdaero, Yoosung-Gu, Daejeon 305-811, Republic of Korea; ^2^Food & Nutrition, Obesity/Diabetes Center, Hoseo University, 165 Sechul-Ri, BaeBang-Yup, Asan-Si, ChungNam-Do 336-795, Republic of Korea

## Abstract

We investigated the antiobesity and hypoglycemic properties of *Prunus mume Sieb. et Zucc *(PMA; Japanese apricot) and *Lithospermum erythrorhizon Sieb. et Zucc* (LES; gromwell) extracts in ovariectomized (OVX) rats that impaired energy and glucose homeostasis. OVX rats consumed either 5% dextrose, 5% PMA extract, 5% LES extract, or 2.5% PMA+2.5% LES extract in the high fat diet. After 8 weeks of treatment, PMA+LES prevented weight gain and visceral fat accumulation in OVX rats by lowering daily food intake and increasing energy expenditure and fat oxidation. PMA+LES prevented the attenuation of leptin and insulin signaling by increasing the expression of leptin receptor in the hypothalamus in OVX rats. PMA+LES significantly reversed the decrease of energy expenditure in OVX rats by increasing expression of UCP-1 in the brown adipose tissues and UCP-2 and UCP-3 in the quadriceps muscles. PMA+LES also increased CPT-1 expression and decreased FAS, ACC, and SREBP-1c in the liver and quadriceps muscles to result in reducing triglyceride accumulation. PMA+LES improved insulin sensitivity in OVX rats. In conclusion, PMA+LES synergistically prevented the impairment of energy, lipid, and glucose metabolism by OVX through potentiating hypothalamic leptin and insulin signaling. PMA+LES may be a useful intervention for alleviating the symptoms of menopause in women.

## 1. Introduction

Obesity is global epidemic that increases the risk of metabolic diseases such as hypertension, type 2 diabetes, dyslipidemia, obstructive sleep apnea, cardiovascular diseases, and certain cancers [1]. Insulin resistance is typically associated with these metabolic diseases and decreasing insulin resistance by losing visceral fat often ameliorates these metabolic disturbances, especially hyperglycemia and hyperlipidemia [1, 2]. In post-menopausal women, estrogen deficiency is associated with obesity, especially with visceral fat accumulation. OVX rats exhibit similar symptoms as post-menopausal women, including increased visceral fat mass and bone loss [3]. In animals ovariectomy leads to increased feed consumption, hyperphagia, and central fat distribution whereas estrogen replacement decreases feed intake throughout the ovarian cycle [4, 5]. Thus, OVX rats fed a high fat diet are a good model for studying the anti-obesity effects of foods.

People attempt to reduce body fat by suppressing appetite and stimulating energy expenditure by taking herbal supplements and functional foods. Appetite is mainly controlled through the hypothalamus, which is a key integrator of nutrient-induced signals of hunger and satiety and is crucial for processing information regarding energy stores and expenditure [6, 7]. Adipokines, especially leptin, convey information about body fat storage to the hypothalamus, and increased leptin signalling decreases food intake and increases energy expenditure resulting in decreased body fat accumulation [6]. However, sustained high leptin levels due to excess body fat induce leptin resistance which impairs regulation of food intake. In addition, hypothalamic energy sensors such as AMP-kinase (AMPK) detect nutrient availability and relays negative feedback signals on food intake [8]. Fat oxidation is regulated by fatty acid transport in mitochondria via carnitine palmitoyltransferase-1 (CPT-1) and by the biosynthesis of fatty acids by acetyl CoA carboxylase (ACC) and fatty acid synthase (FAS) in the cytosol. Thus, obesity might be prevented or reversed by herbs and foods that stimulate fat oxidation and/or suppress food intake.

*Prunus mume Sieb. et Zucc.* (PMA), Japanese apricot, is used in Asian folk medicine for digestive problems [9]. It is also reported to protect against cardiovascular diseases and mumefural, a bioactive compound in Japanese apricot extract, improved blood fluidity in human and animal studies [10]. *Lithospermum erythrorhizon Sieb. et Zucc* (LES), purple gromwell, is an herbal medicine used for inflammatory and infectious diseases [9, 11]. Shikonin, major compound of LES, is an anti-inflammatory and exerts anticancer activity by inducing apoptosis in cancer cells. A recent study demonstrated that shikonin suppresses fat accumulation in 3T3-L1 adipocytes by inhibiting mRNA and protein expressions of peroxisome proliferator-activated receptor-*γ* (PPAR-*γ*), CCAAT/enhancer binding protein-*α* (C/EBP*α*), and sterol regulatory element-binding protein-1c (SREBP-1c) [12]. Our preliminary study also found that water soluble fractions of PMA (50 *μ*g/mL) and LES (50 *μ*g/mL) suppress fat accumulation in 3T3-L1 adipocytes by decreasing PPAR-*γ* activity. However, the anti-obesity effect of LES and/or shikonin has not been investigated in animal or human studies. Therefore, we hypothesized that the long-term administration of PMA and/or LES water extracts would decrease fat accumulation and improve glucose homeostasis in diet-induced obese animals. The present study tested the hypothesis and explored the mechanisms of the anti-obesity action of PMA and LES in ovariectomized (OVX) rats fed a high fat diet.

## 2. Methods and Materials

### 2.1. PMS and LES Water Extract

Dried and ground PMS fruit and LES root (2 kg) were extracted three times by refluxing with water (1 : 5 and then 1 : 3, wt/vol) at 80°C for 3 h, after which the filtered extracts were lyophilized. The yields of PMS fruit and LES root were 21.3 and 25.0%, respectively.

### 2.2. Analysis of Bioactive Compounds

Bioactive components in PMS and LES were analyzed by HPLC using a YMC ODS-AM (250 mm × 4.6 mm I.D.; particle size: 5 *μ*m) reversed-phase column (JASCO-ChromNAV, Japan). For PMS, the mobile phase solvents consisted of 0.1% acetic acid in water and 0.1% acetic acid in acetonitrile with gradient elution with a flow rate of 1.0 mL/min and UV detection was at 280 nm. For LES, the same elution solution was used for isocratic elution and detection was set at 520 nm. The contents of mumefural and shikonin from PMS and LES, respectively, were quantified using each standard as an index compound (Wuxi Gorunjie Natural-Pharma Co., China). Their contents were calculated from each of the standards (2–10 ug/mL) using ChromNAV.

### 2.3. Experimental Animals and Design

Female Sprague-Dawley rats, weighing 220 ± 14 g, had either ovariectomy or sham operations and were housed individually in stainless steel cages in a controlled environment (23°C and with a 12-hour light and dark cycle). All surgical and experimental procedures were performed according to NIH Guidelines and were approved by Hoseo University Animal Care and Use Review Committee. Experimental animals freely consumed water and were assigned their respective diets for the eight-week experimental period. The high fat diet was a modified semipurified AIN-93 formulation [13] consisting of 40 energy percent (En%) carbohydrates, 20 En% protein, and 40 En% fats. The major carbohydrate, protein, and fat sources were starch plus sugar, casein (milk protein), and lard (CJ Co., Seoul).

Sixty OVX rats were randomly divided into four dietary groups: control, PMA, LES, and PMA+LES, containing either 5% dextrose, 5% PMA, 5% LES, or PMA+LES (2.5% each of PMA and LES) in the high fat diet, respectively. The dosage used in the present study is equivalent to approximately 15 g/day for human usage. Fifteen Sham rats had the same diet as ovariectomized control rats. Overnight-fasted serum glucose levels, food and water intake, and body weight were measured every Tuesday at 10 am.

### 2.4. Energy Expenditure by Indirect Calorimetry

After 7 weeks of the assigned treatment, energy expenditure was assessed at the beginning of the dark phase of the light-dark cycle after 6 hours fasting. The rats were placed into the metabolic chambers (airflow = 800 mL/min) with a computer-controlled O_2_ and CO_2_ measurement system (Biopac Systems Inc., Goleta, CA) to determine their calorimetric parameters. The respiratory quotient (RQ) and resting energy expenditure (REE) were calculated using the equations described by Niwa et al. [14]. Average oxygen consumption (VO_2_) and average carbon dioxide production (VCO_2_) were integrated over periods of 30 min. After the experiment, data were averaged over 1 min intervals and VO_2_ and VCO_2_ values were corrected for metabolic body size (Kg^0.75^) [15]. Carbohydrate and fat oxidation were calculated from nonprotein oxygen consumption as were their relative oxidative proportions and the amount of oxygen consumed per gram of substrate oxidized [16].

### 2.5. Oral Glucose Tolerance Test (OGTT) and Liver Glycogen and Triglyceride Contents

Two days after measuring locomotive activity, an OGTT was performed on overnight fasted animals by orally administering 2 g glucose/kg body weight. Blood samples were taken by tail bleeding at 0, 10, 20, 30, 40, 50, 60, 70, 80, 90, and 120 min after glucose loading and serum glucose and insulin were measured with a Glucose Analyzer II (Beckman, Palo Alto, CA) and radioimmunoassay kit (Linco Research, Billerica, MA), respectively. The average of the total areas under the curves for the serum glucose and insulin were calculated by the trapezoidal rule.

After the OGTT, 10 rats were freely provided with food and water for 2 days and the next day they were deprived of food for 16 hours and blood was collected for further analysis. The rats were then anesthetized with a mixture of ketamine and xylazine, and human regular insulin (5 U/kg body weight) was injected through the inferior vena cava. Ten min later, the rats were killed by decapitation and tissues were rapidly collected, frozen in liquid nitrogen, and stored at −70°C for further experiments. Glycogen contents in the livers were determined by centrifuging liver lysates at 4000 ×g for 10 min after which supernatants were deproteinized with 1.5 N perchloric acid. The glycogen content was calculated from glucose concentrations derived from glycogen hydrolyzed by *α*-amyloglucosidase in an acid buffer [17]. Triacylglycerol was extracted with chloroform-methanol (2 : 1, vol/vol) from the livers and resuspended in pure chloroform [18]. After evaporating the chloroform, the residues were suspended with PBS with 0.1% triton X-100 and the suspension was sonicated and boiled for 5 min. The triacylglycerol contents of the suspensions were assayed using a Trinder kit (Asan Pharm., Seoul, Korea). Serum triglyceride and total cholesterol were determined using the same kits as for liver, and serum leptin levels were determined using a radioimmunoassay kit (Linco Research).

### 2.6. Immunoblot Analysis

Frozen hypothalami of six rats were lysed with a 20 mM Tris buffer (pH 7.4) containing 2 mM EDTA, 137 mM NaCl, 1% NP40, 10% glycerol, and 12 mM *α*-glycerol phosphate and protease inhibitors. Lysates containing equal amounts of protein (30–50 *μ*g) were resolved by SDS-PAGE, and immunoblotting was performed with specific antibodies against phosphorylated signal transducer and activator of transcription (STAT-3)^tyr705^, Akt^ser473^ and AMPK kinase (AMPK)^thr172^, and *β*-actin. The intensity of protein expression was determined using Imagequant TL (Amersham Biosciences, Piscataway, NJ). Three sets of two samples per each group were determined (*n* = 6).

### 2.7. RNA Isolation and Reverse Transcription Polymerase Chain Reaction (RT-PCR)

The liver, quadricep muscles, and brown adipose tissue from four rats from each group were collected at the end of treatment. Total RNA was isolated from the liver using a monophasic solution of phenol and guanidine isothiocyanate (Trizol reagent, Gibco-BRL, Rockville, MD), followed by extraction and precipitation with isopropyl alcohol [19]. The cDNA was synthesized from equal amounts of total RNA with superscript III reverse transcriptase, and polymerase chain reaction (PCR) was performed with high fidelity Taq DNA polymerase. Equal amounts of cDNA were mixed with SYBR green mix and they were analyzed by a realtime PCR machine (BioRad, Richmond, CA). The expression level of the gene of interest was corrected for that of the house keeping gene, *β*-actin. The primers used to detect rat CPT-1, ACC, SREBP-1c, FAS, uncoupling protein (UCP)-1, UCP-2, UCP-3, and *β*-actin genes were described previously [19].

### 2.8. Statistical Analysis

Statistical analysis was performed using the SAS software and all results are expressed as a mean ± standard deviation. The metabolic effects of PMA, LES, PMA+LES, and the control were determined by a one-way analysis of variance (ANOVA). Significant differences in the main effects among the groups were identified by Tukey's test at *P* < 0.05. The differences between the OVX rats and Sham rats were determined by two-sample *t*-test.

## 3. Results

### 3.1. The Contents of Mumefural and Shikonin

Mumefural and shikonin were used as indicator compounds for the degrees of extraction of PMS and LES, respectively. The concentrations of mumefural were 8.4 ± 1.2 mg/100 g dry PMS (*n* = 3) whereas those of shikonin were 106.5 ± 19.3 mg/100 g dry LES (*n* = 3).

### 3.2. Body Weight and Energy Balance

**Figure 1:**
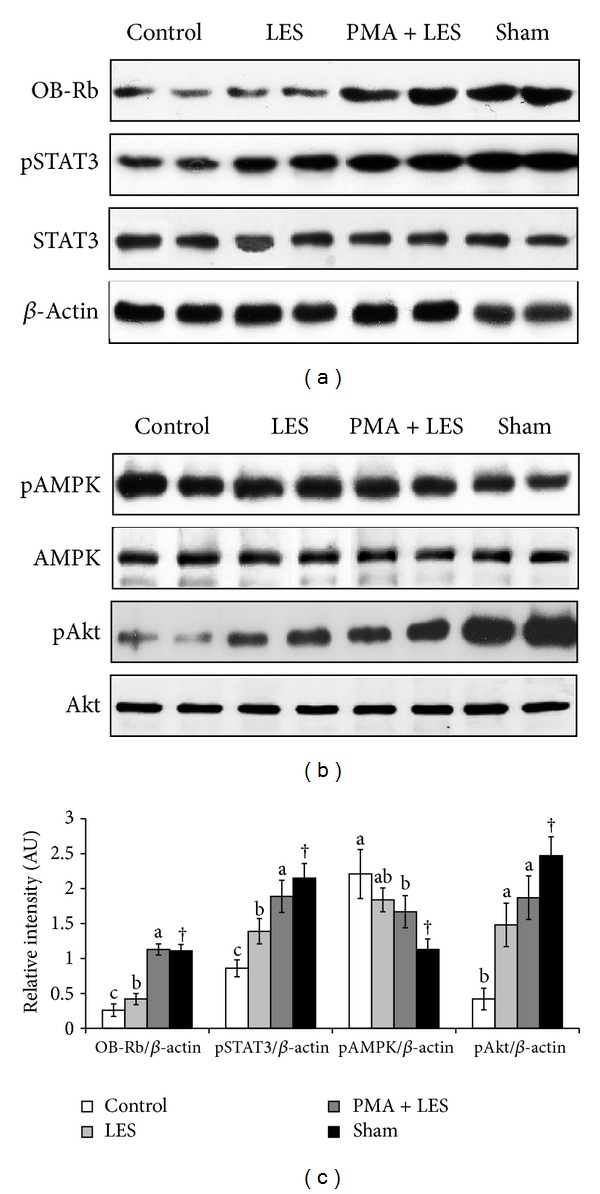
Phosphorylation of STAT3, AMPK, and Akt in the hypothalamus. Control, OVX rats fed high fat diet (HFD) with 5% cellulose; PMA, OVX rats fed HFD with 5% *Prunus mume*; LES, OVX rats fed HFD and 5% *Lithospermum erythrorhizon*; PMA+LES, OVX rats fed HFD with 2.5% PMA and 2.5% LES. Sham, Sham rats fed HFD with 5% cellulose. The intensity of phosphorylation of STAT3, AMPK, and Akt proteins was corrected by that of respective total unphosphorylated proteins to be expressed as relative intensity (arbitrary unit). *Significantly different among the groups of OVX rats at *P* < 0.05  (*n* = 6).  ^a,b^Different superscripts on the bars of each variable indicate significant differences in each variable. ^†^Significant difference between OVX rats and Sham rats.

OVX rats gained significantly more body weight and visceral fat (periuterine fat and retroperitoneal fat) than sham rats. PMA and LES suppressed the increases in body weight and visceral fat mass in OVX rats, but PMA+LES combined suppressed them more than either individually, with increases in visceral fat mass and body weight that were similar to the Sham rats (Table 1). Serum leptin levels were not significantly different among any of the groups despite the differences in visceral fat (Table 1). The body weight and visceral fat contents were modulated by the balance of energy intake and energy expenditure. Energy intake was slightly, but not significantly, higher in OVX rats. PMA+LES decreased food intake in OVX rats to less than Sham rats (Table 1). This might be related to leptin signaling in the hypothalamus. OVX rats lower expression levels of long-form leptin receptor (OB-Rb) in comparison to the Sham rats and PMA+LES restored it to similar levels as Sham rats (Figure 1). In consistency with the levels of OB-Rb, OVX rats had less phosphorylation of STAT-3 and Akt than Sham rats and increased phosphorylation of AMPK (Figure 1). LES and PMA+LES increased the phosphorylation of STAT-3 and Akt and PMA+LES resulted in equal phosphorylation of STAT-3 as that of Sham rats (Figure 1). In addition, the phosphorylation of AMPK tended to decrease with PMA+LES in OVX rats in comparison to the control, but it was not significantly different (Figure 1).

Energy expenditure was significantly lower in OVX rats than Sham rats (Table 2). LES elevated daily energy expenditure and it was synergistically increased by PMA+LES (Table 2). Respiratory quotient tended to be higher in OVX rats than Sham rats and all treatments modified respiratory quotient in OVX rats. Carbohydrate oxidation was higher in OVX rats than Sham rats but none of the treatments affected carbohydrate oxidation. By contrast, fat oxidation was lower in OVX rats and in OVX rats, and fat oxidation was increased in the ascending order of control <LES = PMA< PMA+LES (Table 2). Thus, PMA+LES suppressed fat accumulation in OVX rats by increasing both energy expenditure and fat oxidation.

### 3.3. Serum Lipid Profiles

Total and LDL cholesterol and triglyceride concentrations were significantly higher and HDL cholesterol was lower in OVX rats than Sham rats (Table 3). PMA alone did not improve serum lipid profiles (Table 3). However, LES decreased circulating total and LDL cholesterol and triglyceride levels. Serum HDL cholesterol was not affected by either PMA or LES alone (Table 3). PMA+LES synergistically lowered serum total and LDL cholesterol and triglyceride concentrations and raised HDL concentrations (Table 3).

### 3.4. Glucose Homeostasis

Overnight-fasted serum glucose levels were also higher in OVX rats in comparison to Sham rats, but PMA, LES, or PMA+LES had no significant effect on serum glucose levels (Table 3). The increased serum glucose levels were due to increased insulin resistance, not serum insulin levels. Overnight-fasted serum insulin levels were much higher in OVX rats than Sham rats and were lower with PMA and LES and lowest with PMA+LES administration (Table 3). Homeostatic model assessment of insulin resistance (HOMA-IR), an indicator of insulin resistance, was much higher in OVX rats than Sham rats. HOMA-IR was decreased in a descending order of control >PMA = LES> PMA+LES (Table 3).

**Figure 2:**
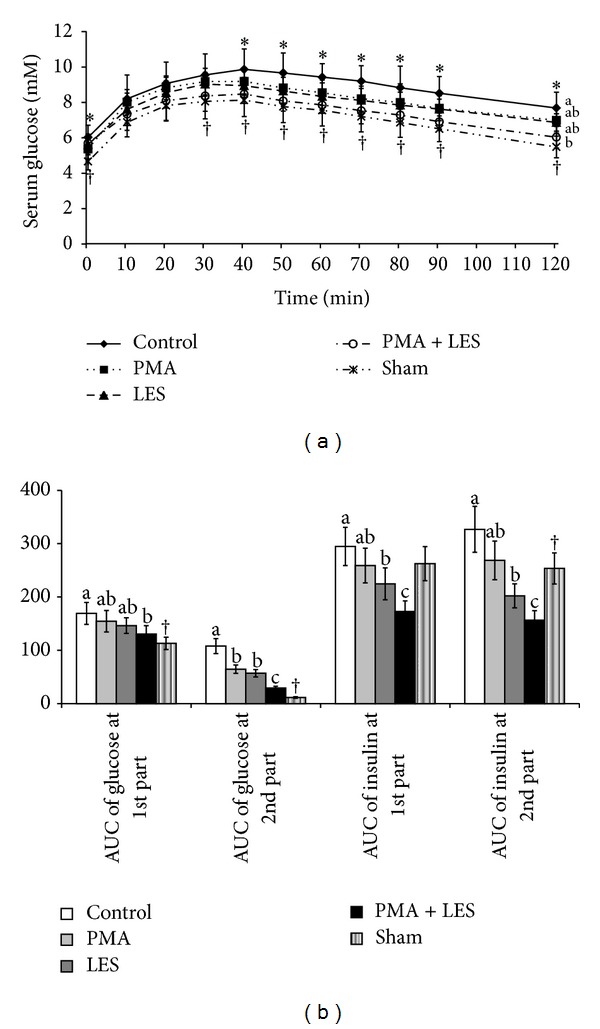
Serum glucose levels and area under the curve (AUC) of serum glucose and insulin during oral glucose tolerance test (OGTT). Control, OVX rats fed high fat diet (HFD) with 5% cellulose; PMA, OVX rats fed HFD with 5% *Prunus mume*; LES, OVX rats fed HFD and 5% *Lithospermum erythrorhizon*; PMA+LES, OVX rats fed HFD with 2.5% PMA and 2.5% LES. Sham, Sham rats fed HFD with 5% cellulose. (a) Changes of serum glucose levels during OGTT. (b) The average of the AUC of glucose and insulin during the first part (0–40 min) and second part (40–120 min) of OGTT. Each dot and bar represents the mean ± SD (*n* = 15). *Significantly different among the groups of OVX rats at *P* < 0.05.  ^a,b^Different superscripts on the bars of each variable indicate significant differences in each variable. ^†^Significant difference between OVX rats and Sham rats.

OVX rats exhibited impaired glucose intolerance during the OFTT as compared to Sham rats. PMA and LES slightly improved glucose tolerance by decreasing serum glucose levels rapidly after reaching the peak levels (Figure 2(a)), and the PMA+LES group exhibited lower peak serum glucose levels and a more rapid decrease from the peak. Glucose tolerance of PMA+LES treated rats was similar to Sham rats (Figure 2(a)). Area under the curve (AUC) of glucose at the first part (0–40 min) and second part (40–120 min) of OGTT was higher in the OVX than Sham rats (Figure 2(b)). In OVX rats, PMA+LES significantly lowered the AUC of glucose at the first part and the AUC of glucose at the second part was lowered in the descending order of the control >PMA = LES> PMA+LES (Figure 2(b)). These differences were related to insulin secretion and insulin sensitivity: AUC of insulin during the first and second part of OGTT was higher in OVX rats than Sham rats. LES decreased AUC of insulin at the first and second part and PMA+LES decreased it further (Figure 2(b)). These results suggest that LES and PMA normalized glucose tolerance by improving insulin sensitivity and insulin secretion, respectively.

### 3.5. Hepatic and Skeletal Muscle Glucose Metabolism

**Figure 3:**
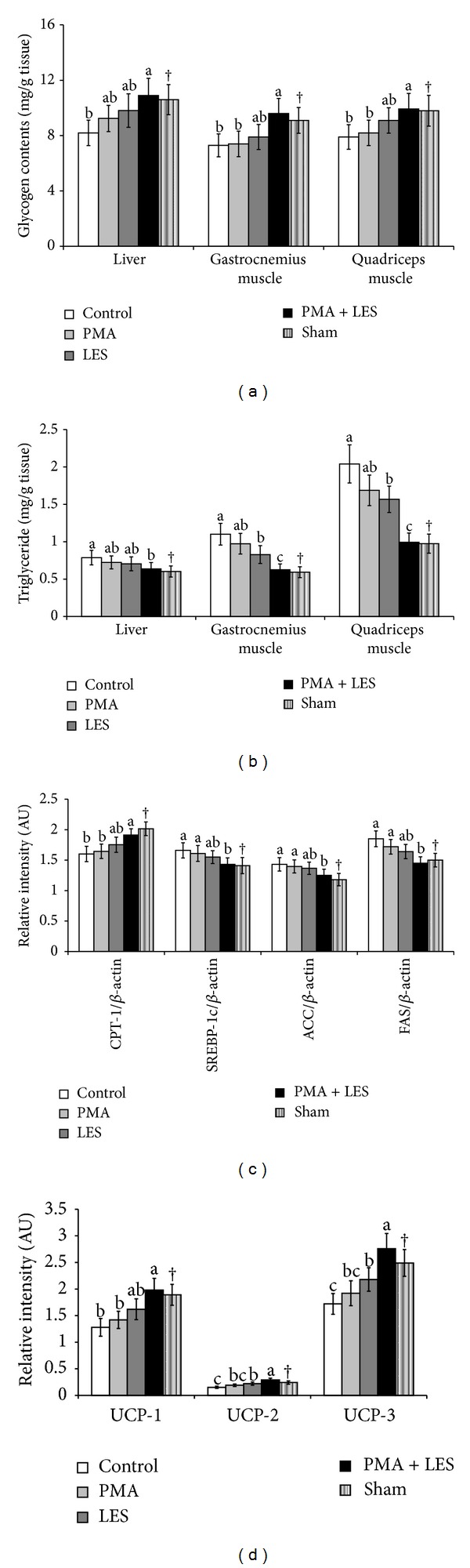
Glucose and lipid metabolism in the liver and skeletal muscles. Control, OVX rats fed high fat diet (HFD) with 5% cellulose; PMA, OVX rats fed HFD with 5% *Prunus mume*; LES, OVX rats fed HFD and 5% *Lithospermum erythrorhizon*; PMA+LES, OVX rats fed HFD with 2.5% PMA and 2.5% LES. Sham, Sham rats fed HFD with 5% cellulose. (a) Stored glycogen levels. (b) Triglyceride accumulation. (c) mRNA expression of CPT-1, SREBP-1c, FAS, and ACC in the liver. (d) mRNA expression of UCP-1 in the brown adipose tissues and UCP-2 and UCP-3 in the quadriceps muscles. *Significantly different among the groups of OVX rats at *P* < 0.05.   ^a,b^Different superscripts on the bars of each variable indicate significantly differences in each variable. ^†^Significant difference between OVX rats and Sham rats.

Glycogen storage in the liver and gastrocnemius and quadriceps muscles was lower in OVX rats than Sham rats (Figure 3(a)). PMA+LES prevented the decrease of glycogen storage in liver and skeletal muscles of OVX rats and the glycogen levels of OVX rats were similar to Sham rats (Figure 3(a)). Triglyceride storage in the liver and gastrocnemius and quadriceps muscles were higher in OVX rats than Sham rats. LES and PMA+LES decreased the triglyceride storage in the liver and skeletal muscles with levels in PMA+LES reaching those of Sham rats (Figure 3(b)).

The expression of hepatic CPT-1, the mitochondrial transporter of fatty acids, was lower and the expression of hepatic SREBP-1c, FAS, and ACC, related to fatty acid synthesis, was higher in OVX rats compared to Sham rats (Figure 3(c)). In OVX rats LES and especially PMA+LES increased the expression of CPT-1 and decreased the expression of FAS, SREBP-1c, and ACC (Figure 3(c)) with expressions in PMA+LES similar to those of Sham rats. Their expression in quadriceps muscle was similar to that of the liver (data not shown). Expression of UCP-2 and UCP-3 in the quadriceps muscle was lower in OVX rats compared to Sham rats, but PMA+LES prevented the decrease (Figure 3(d)). In brown adipose tissues, the expression of UCP-1 was lower in OVX compared to Sham rats, and PMA+LES reversed the decrease (Figure 3(d)).

## 4. Discussion

Menopause presents metabolic challenges that include weight gain characterized by increased visceral fat resulting in metabolic diseases such as diabetes, hypertension, dyslipidemia and osteoporosis [1]. Since energy balance is maintained by regulating energy intake and expenditure, estrogen may affect both phenomena. Hormone replacement therapy (HRT) inhibits the increase in post-menopausal fat mass by about 60% and concomitantly decreases cardiovascular risks [20, 21]. However, HRT use is limited since it has adverse effects such as increased risk of breast and endometrial cancers [22]. The present study revealed that PMA+LES synergistically prevented the impairment of energy, glucose, and lipid regulation in OVX rats, and the regulation in PMA+LES treated OVX rats was similar to Sham rats. PMA+LES prevented body weight and visceral fat accumulation by normalizing daily energy expenditure and fat oxidation in OVX rats. The increased energy expenditure was associated with increased expression of UCP-2 and UCP-3 in the skeletal muscles. PMA+LES also improved serum lipid profiles and lowered triglyceride accumulation in the liver and skeletal muscle. Fat metabolism was also improved by enhancing fat oxidation and decreasing fat synthesis: PMA+LES increased CPT-1 expression and decreased ACC, FAS, and SREBP-1c in the liver and skeletal muscles. PMA+LES improved insulin resistance as evidenced by decreased serum insulin levels. Therefore, PMA+LES prevented the dysregulation of energy, lipid, and glucose metabolism by OVX, suggesting it may be useful for alleviating the metabolic disturbances associated with menopause.

Estrogen deficiency increases the accumulation of body fat, especially abdominal fat, in animals and humans, but is reversible by estrogen therapy [23–25]. Although body fat accumulation is related to energy imbalance, the loss of ovarian function promotes a diet-independent increase in adiposity [24]. Several studies have found that ovariectomized animals have lower energy expenditure, without corresponding decreases in energy intake, resulting in adipocyte hypertrophy, adipose tissue inflammation, and the development of fatty liver [26–29]. However, the effects of estrogen insufficiency on energy intake remain controversial [30–32]. Some studies have shown that OVX increases energy intake [30, 31], whereas others did not [33]; however, estrogen replacement restores normal energy metabolism [30, 31]. In the estrous cycle, higher estrogen levels normally decrease energy intake and increase energy expenditure resulting in weight loss, but the response to rising estrogen levels is delayed and ineffective in obese animals [33].

The changes in energy expenditure in OVX rats might be related to increased thermogenesis by estrogen. A single injection of estrogen increases heat production by increasing UCP-1, UCP-2, and UCP-3 in adipose tissues and skeletal muscles without changing food intake in OVX sheep; however, long-term estrogen treatment decreased food intake but exhibited no thermogenesis in OVX sheep [28]. In addition to decreased basal energy expenditure, ovariectomized mice exhibit decreased physical activity [29]. The present study showed that PMA+LES increased UCP-2 and UCP-3 in the skeletal muscle in OVX rats and UCP-1 in the brown adipose tissues, which might have contributed to the elevated energy expenditure, which was similar to Sham rats.

The mechanism of how estrogen regulates energy balance is poorly understood. Energy intake is regulated by leptin signaling in the hypothalamus. However, estrogenic effects on leptin secretion and signaling are controversial. Some studies have reported that OVX-induced weight gain is associated with increased circulating leptin and central leptin resistance [34], but other studies have shown that estrogen deficiency suppresses leptin secretion, [35, 36] which is consistent with this study. Experimental evidence shows that estrogen modulates fat accumulation by binding to estrogen receptor-*α* [37, 38]. The estrogen exerts leptin-like activity through activation of intracellular signals in hypothalamic melanocortin cells resulting in decreased food intake, increased energy expenditure, and weight loss [39]. Rats with overiectomy or deletion or siRNA-mediated silencing of estrogen receptor-*α* in the hypothalamus develop leptin resistance that attenuates leptin signaling in the hypothalamus and results in obesity [38, 40, 41]. Estrogen replacement in ovariectomized animals suppresses the development of obesity by decreasing food intake and increasing energy expenditure [40]. Therefore, the efficacy of PMA+LES could be directly associated with the potentiation of estrogen receptors, but that was not addressed in this study. The direct actions of PMA+LES on the estrogen receptor need to be studied.

The present study showed that OVX decreased OB-Rb in the hypothalamus and attenuated leptin signaling despite a lack of changes in serum leptin levels. PMA+LES prevented the decrease of OB-Rb and improved leptin signaling by potentiating the phosphorylation of STAT3 in the hypothalamus. This might be related to estrogen effects on OB-RB expression and leptin secretion. Consistent with the potentiated hypothalamic leptin signaling, PMA+LES suppressed the increase in food intake in OVX rats that tended to increase food intake.

PMA+LES may also affect fat utilization. Lee et al. [12] have shown that shikonin, a major component of LES, lowered fat accumulation in 3T3-L1 adipocytes by decreasing the expression of PPAR-*γ*, C/EBP*α*, and SREBP-1c which are involved in fat synthesis. This study also showed that PMA+LES elevated CPT-1 and lowered ACC, SREBP-1c, and FAS in the liver and skeletal muscles, which might be responsible for the lowered fat mass in OVX rats. This may be related to the improved insulin sensitivity with PMA+LES. The improvement markedly decreased serum insulin levels, which suppresses lipid synthesis. Thus, PMA+LES contained ingredients besides shikonin which activates oxidation and suppresses synthesis of fatty acids. In the present study, OVX rats had slightly, but not significantly, higher energy intakes than Sham rats but decreased energy expenditure, which was mostly responsible for the weight gain and was prevented by PMA+LES.

In conclusion, OVX induced fat accumulation in the viscera, liver, and skeletal muscles, mostly due to decreased energy expenditure. The increased fat accumulation exacerbated insulin resistance and impaired glucose and lipid metabolism. PMA+LES treatment in OVX rats normalized fat accumulation by decreasing food intake and increasing energy expenditure through potentiating hypothalamic insulin signaling and restoring normal glucose and lipid metabolism. Therefore, PMA+LES may be useful for alleviating metabolic dysregulation in post-menopausal women.

## Figures and Tables

**Table 1:** Metabolic parameters at the end of experimental periods.

	Control (*n* = 15)	PMA (*n* = 15)	LES (*n* = 15)	PMA+LES (*n* = 15)	Sham (*n* = 15)
Body weight (g)	284.2 ± 18.6^a^	275.5 ± 22.7^ab^	270.8 ± 17.1^b^	255.1 ± 15.5^c∗^	251.3 ± 11.2^†^
Body weight gain (g)	63.5 ± 4.8^a^	55.1 ± 4.7^b^	50.2 ± 4.3^b^	38.2 ± 2.7^c∗^	35.2 ± 2.4^†^
Peri-uterine fat (g)	7.8 ± 0.9^a^	6.4 ± 0.8^b^	5.9 ± 0.7^b^	5.1 ± 0.8^c∗^	5.3 ± 0.7^†^
Ratio of peri-uterine fat and body weight	0.027 ± 0.005^a^	0.023 ± 0.004^b^	0.022 ± 0.004^b^	0.020 ± 0.004^b∗^	0.021 ± 0.003^†^
Retroperitoneum fat (g)	5.9 ± 0.9^a^	4.1 ± 0.7^b^	3.8 ± 0.6^b^	3.1 ± 0.7^c∗^	3.0 ± 0.4^†^
Ratio of retroperitoneum fat and body weight	0.021 ± 0.003^a^	0.015 ± 0.003^b^	0.01 ± 0.003^b^	0.012 ± 0.003^b∗^	0.012 ± 0.003
Caloric intakes (kcal/day)	130.9 ± 15.9^a^	128.2 ± 15.5^a^	126.6 ± 14.2^ab^	115.8 ± 13.4^b∗^	120.5 ± 15.6
Overnight fasted leptin levels (ng/mL)	3.4 ± 0.6	3.8 ± 0.6	3.7 ± 0.6	3.7 ± 0.6	4.0 ± 0.8^†^

Control, OVX rats fed a high fat diet (HFD) with 5% cellulose; PMA, OVX rats fed HFD with 5% *Prunus mume*;  LES, OVX rats fed HFD 5% *Lithospermum erythrorhizon; *PMA+LES, OVX rats fed HFD with 2.5% PMA and 2.5% LES. Sham, Sham rats fed a high fat diet (HFD) with 5% cellulose.

*Significantly different among the groups of OVX rats at *P* < 0.05.

^a,b,c^Values on the same row with different superscripts were significantly different at *P* < 0.05.

^†^Significant difference between OVX rats and Sham rats at *P* < 0.05.

**Table 2:** The parameters of indirect calorimetry at the end of experiment.

	Control (*n* = 15)	PMA (*n* = 15)	LES (*n* = 15)	PMA+LES (*n* = 15)	Sham (*n* = 15)
Energy expenditure (kcal/kg^0.75^/day)	113.5 ± 14.8^b^	121.8 ± 14.4^ab^	128.8 ± 14.7^a^	139.6 ± 15.1^a∗^	137.0 ± 14.8^†^
Respiratory quotient	0.86 ± 0.10	0.84 ± 0.09	0.85 ± 0.09	0.82 ± 0.09	0.81 ± 0.09
VO_2_ (mL/kg^0.75^/min)	16.2 ± 1.9^c^	17.4 ± 2.0^b^	18.4 ± 2.1^b^	19.9 ± 2.2^a∗^	19.6 ± 2.1^†^
VCO_2_ (mL/kg^0.75^/min)	14.0 ± 1.7	14.6 ± 1.8	15.6 ± 1.9	16.3 ± 1.9	15.9 ± 1.8
Carbohydrate oxidation (mg/kg^0.75^/min)	6.4 ± 0.9	5.8 ± 0.8	6.6 ± 0.9	5.8 ± 0.8	5.1 ± 0.8^†^
Fat oxidation (mg/kg^0.75^/min)	5.5 ± 0.8^c^	7.1 ± 1.0^b^	7.1 ± 1.0^b^	9.1 ± 1.4^a∗^	9.5 ± 1.2^†^

Control, OVX rats fed a high fat diet (HFD) with 5% cellulose; PMA, OVX rats fed HFD with 5% *Prunus mume*;  LES, OVX rats fed HFD 5%* Lithospermum erythrorhizon*; PMA+LES, OVX rats fed HFD with 2.5% PMA and 2.5% LES. Sham, Sham rats fed a high fat diet (HFD) with 5% cellulose.

*Significantly different among the groups of OVX rats at *P* < 0.05.

^a,b,c^Values on the same row with different superscripts were significantly different at *P* < 0.05.

^†^Significant difference between OVX rats and Sham rats at *P* < 0.05.

**Table 3:** Serum lipid and glucose profiles in overnight fasted rats.

	Control (*n* = 15)	PMA (*n* = 15)	LES (*n* = 15)	PMA+LES (*n* = 15)	Sham (*n* = 15)
Total cholesterol (mg/dL)	109.6 ± 10.9^a^	99.6 ± 10.8^ab^	91.2 ± 10.9^b^	83.6 ± 9.1^c∗^	88.9 ± 9.3^†^
LDL cholesterol (mg/dL)	72.9 ± 6.8^a^	63.8 ± 7.0^a^	57.7 ± 7.1^b^	55.8 ± 6.6^b∗^	53.7 ± 6.8^†^
HDL cholesterol (mg/dL)	16.1 ± 2.6^b^	16.9 ± 2.7^b^	18.5 ± 2.6^ab^	19.3 ± 2.4^a∗^	18.7 ± 2.1^†^
Triglyceride (mg/dL)	102.9 ± 10.3^a^	94.2 ± 9.8^a^	68.8 ± 9.6^b^	53.7 ± 8.6^c∗^	82.5 ± 9.1^†^
Glucose levels (mg/dL)	108.6 ± 13	99.4 ± 13.3	102.0 ± 15.1	100.5 ± 12.8	83.6 ± 11.7^†^
Insulin levels (ng/mL)	1.37 ± 0.28^a^	1.09 ± 0.25^b^	0.98 ± 0.17^b^	0.79 ± 0.14^c∗^	1.07 ± 0.21^†^
HOMA-IR	8.2 ± 0.9^a^	6.2 ± 0.8^b^	5.6 ± 0.8^b^	4.4 ± 0.7^c∗^	4.9 ± 0.7^†^

Control, OVX rats fed a high fat diet (HFD) with 5% cellulose; PMA, OVX rats fed HFD with 5% *Prunus mume*; LES, OVX rats fed HFD 5%* Lithospermum erythrorhizon*; PMA+LES, OVX rats fed HFD with 2.5% PMA and 2.5% LES. Sham, Sham rats fed a high fat diet (HFD) with 5% cellulose.

*Significantly different among the groups of OVX rats at *P* < 0.05.

^a,b,c^Values on the same row with different superscripts were significantly different at *P* < 0.05.

^†^Significant difference between OVX rats and Sham rats at *P* < 0.05.
